# U.S. adults are not meeting recommended levels for fish and omega-3 fatty acid intake: results of an analysis using observational data from NHANES 2003–2008

**DOI:** 10.1186/1475-2891-13-31

**Published:** 2014-04-02

**Authors:** Yanni Papanikolaou, James Brooks, Carroll Reider, Victor L Fulgoni

**Affiliations:** 1 Nutritional Strategies Inc, 59 Marriot Place, Paris, ON N3LOA3, Canada; 2 Pharmavite, LLC, Northridge, CA, USA; 3 Pharmavite, LLC, Mission Hills, CA, USA; 4 Nutrition Impact, LLC, Battle Creek, MI, USA

**Keywords:** NHANES, Usual intake, Fish, Omega-3 fatty acids, EPA, DHA, Cardiovascular

## Abstract

**Background:**

The American Heart Association’s Strategic Impact Goal Through 2020 and Beyond recommends ≥ two 3.5-oz fish servings per week (preferably oily fish) partly to increase intake of omega-3 fatty acids eicosapentaenoic acid (EPA) and docosahexaenoic acid (DHA). We examined the intake of total fish, fish high in omega-3 fatty acids, α-linolenic acid, EPA, and DHA in U.S. adults (19 + years) using data from the National Health and Nutrition Examination Survey, 2003–2008.

**Methods:**

Usual intakes from foods alone and from foods plus dietary supplements were determined using the methods from the National Cancer Institute.

**Results:**

Mean usual intake of total fish and fish high in omega-3 fatty acids was 0.61 ± 0.03 and 0.15 ± 0.03 oz/day, 0.43 and 0.07 respectively. Total fish and fish high in omega-3 fatty acids median intake was 0.43 and 0.07 oz/day, respectively. Intake from foods alone for ALA, EPA and DHA was 1.5 ± 0.01 g/d, 23 ± 7 mg/d and 63 ± 2 mg/d, respectively. ALA, EPA and DHA from food only median intakes were 1.4 g/d, 18 mg/d and 50 mg/d, respectively. Intake of ALA, EPA and DHA from foods and dietary supplements was 1.6 ± 0.04 g/d, 41 ± 4 mg/d and 72 ± 4 mg/d, respectively. While intakes of fish high in omega-3 fatty acids were higher in older adults (0.13 ± 0.01 oz/d for those 19–50 yrs and 0.19 ± 0.02 oz/d for those 51+ year; p < 0.01) and in males as compared to females (0.18 ± 0.02 vs 0.13 ± 0.01 oz/d, respectively; p < 0.05), few consumed recommended levels. Males also had higher (p < 0.05) intake of EPA and DHA from foods and dietary supplements relative to females (44 ± 6 vs 39 ± 4 and 90 ± 7 vs 59 ± 4 mg/d, respectively) and older adults had higher intakes of EPA, but not DHA compared to younger adults (EPA: 34 ± 3 vs 58 ± 9, p < 0.05; DHA: 68 ± 4 vs 81 ± 6, p < 0.05).

**Conclusions:**

As omega-3 fatty acids are deemed important from authoritative bodies, supplementation in addition to food sources may need to be considered to help U.S. adults meet recommendations.

## Introduction

Major advances have been made in the prevention, diagnosis, and treatment of cardiovascular disease (CVD) over the last four decades. This has contributed to a reduced mortality, however, morbidity and mortality from CVD remains high with prevalence estimates in the United States at approximately 82.6 million. In 2008, heart disease and cerebrovascular disease were the first and fourth leading causes of death, respectively. The total cost of CVD is an economic burden on health care with an estimated total cost of $298 million [1]. The prevention of CVD has become a public health initiative with many attributing variables, of which includes the consumption of fish and fish-derived omega-3 fatty acids [2-4]. Both randomized clinical interventions and observational studies highlight the cardio-protective effects associated with diets higher in fish and omega-3 fatty acid intake, particularly the longer-chain fatty acids, eicosapentaenoic acid (EPA) and docosahexaenoic acid (DHA). These studies have prompted the 2010 Dietary Guidelines for Americans to recommend the consumption of two servings of seafood per week (4 oz per serving), to provide an average of 250 mg per day of long-chain omega-3 fatty acids, in persons with and without CVD [5]. The American Heart Association’s Strategic Impact Goal Through 2020 and Beyond recommends at least two 3.5-oz fish servings per week, with an emphasis on oily fish, to increase intake of omega 3-fatty acids EPA and DHA [6].

Long-chain omega-3 fatty acids may reduce CVD risk through several mechanisms, of which include lowering effects on lipids, inflammatory markers, and platelets [7]. A recent systematic review limited to cardiovascular events in randomized controlled trials and clinical trials demonstrated several cardiovascular favorable effects when marine omega-3 fatty acids were provided as food or in a supplement for a minimum of six months duration [4]. While the cardioprotective effect of ALA has been questioned previously [8], several studies using large sample populations have reported ALA intake to be inversely associated with primary cardiovascular events [9].

Since a substantiated benefit has been well-established for fish and omega-3 fatty acid consumption, it becomes important to assess usual intakes in American adults. Therefore, the objective of the present analysis was to examine the intake of total fish, fish high in omega -3 fatty acids, α-linolenic acid (ALA), eicosapentaenoic acid (EPA), and docosahexaenoic acid (DHA) in U.S. adults (19 + years of age) using data from the National Health and Nutrition Examination Survey, 2003–2008.

## Subjects and methods

### Study population

The NHANES is a nationally representative, cross-sectional survey of non-institutionalized, civilian U.S. residents, collected by the National Center for Health Statistics of the Centers for Disease Control and Prevention [10]. Written informed consent was obtained for all participants or proxies, and the survey protocol was approved by the Research Ethics Review Board at the National Center for Health Statistics. Data from NHANES 2003–2004, 2005–2006, 2007–2008 were combined for these analyses [11,12]. The nutrient intakes for NHANES 2003–2008 are from the USDA Food and Nutrient Database for Dietary Studies 3.0 [13]. The combined sample included 14,338 participants, aged **≥**19 years of age, who had complete 24-h dietary intake data from What We Eat in America [14]. Subjects <19 y of age and pregnant and/or lactating women were excluded.

### Methods and statistical analysis

Total fish and fish high in omega-3 fatty acids were derived from the MyPyramid Database (version 2.0) [15]. In this database USDA provided ounce equivalents of total fish and fish high in omega-3 fatty acids for each product consumed, which were then totaled for each day of recorded intake for each subject.

Estimate mean usual intakes of total fish and fish high in omega-3 fatty acids were determined using the NCI method [16]. The usual intake of omega-3 fatty acids from foods alone and from foods plus dietary supplement use were also determined using the NCI method [16]. Dietary Reference Intakes age groups [17] were used to establish percentile rankings of nutrient intakes to compare against recommendations. Covariates used in the NCI model were survey day (one or two) and a weekend day flag (Friday/Saturday/Sunday vs. others) as covariates. Complete details of the NCI method are shown elsewhere [16], and the SUDAAN/SAS macros (SAS, version 9; SAS Institute Inc) necessary to fit this model and to perform the estimation of usual intake distributions are available on the NCI website [18].

All statistical analyses were performed using SUDAAN software (version 10.0, Research Triangle Park, NC). Survey weights were used to generate nationally representative estimates for the US population and adjusted for the complex sample design of NHANES. Data are presented as means ± standard errors and a more conservative p-value of <0.05 was set to establish significance due to the large sample size of the NHANES analyses.

## Results

### Total fish and fish high in omega-3 fatty acids

Mean usual intake of total fish and mean usual intake of fish high in omega-3 fatty acids were calculated for several age categories in adults ≥19 years of age (see Tables 1 and 2). Mean usual intake of total fish and fish high in omega-3 fatty acids was 0.61 ± 0.03 and 0.15 ± 0.03 oz equivalents/day, respectively. Older adults (≥51 years of age) had greater total fish and fish high in omega-3 fatty acid intake in comparison to the younger adults (19–50 years of age; 0.66 ± 0.04 vs. 0.58 ± 0.03, p < 0.05). Males of all ages had greater mean usual intake of total fish and fish high in omega-3 fatty acids relative to the females of comparable ages (see Tables 1 and 2). While intakes of EPA and DHA were higher in older adults (0.13 ± 0.01 oz/d for those 19–50 yrs and 0.19 ± 0.02 oz/d for those 51+ year; p < 0.01) and in males as compared to females (0.18 ± 0.02 vs. 0.13 ± 0.01 oz/d, respectively; p < 0.05), few are consuming recommended levels.

**Table 1:** Mean usual intake of total fish in adults from NHANES 2003–2008 (oz equivalents/day)

			**Usual intake**		**Percentile**				
**Gender**	**Age**	**N**	**Mean**	**SE**	**10**	**25**	**50**	**75**	**90**
All	19 + Years	14,338	0 61	0.03	0.11	0.22	0.43	0.80	1.33
All	19 - 50 Years	7,585	0.58	0.03	0.10	0.20	0.39	0.75	1.27
All	51 + Years	6,753	0.66	0.04	0.13	0.25	0.48	0.88	1.41
Male	19 + Yeats	7,302	0.71	0.04	0.11	0.23	0.48	0.95	1.62
Male	19 - 50 Years	3,944	0.69	0.04	0.11	0.22	0.47	0.93	1.59
Male	51 + Years	3,358	0.74	0.06	0.12	0.24	0.51	1.00	1.67
Female	19 + Years	7,036	0.51	0.03	0.11	0.21	0.39	0.69	1.08
Female	19 - 50 Years	3,641	0.45	0.03	0.10	0.18	0.34	0.60	0.96
Female	51 + Years	3,395	0.59	0.05	0.14	0.26	0.47	0.80	1.21

**Table 2:** Mean usual intake for fish high in Omega-3 fatty acids in adults from NHANES 2003–2008 (oz equivalents/day)

			**Usual intake**		**Percentile**				
**Gender**	**Age**	**N**	**Mean**	**SE**	**10**	**25**	**30**	**75**	**90**
All	19 + Years	14,338	0.15	0.01	0.01	0.03	0.07	0.17	0.38
All	19 - 50 Years	7,585	0.13	0.01	0.01	0.02	0.06	0.14	0.32
All	51 + Years	6,753	0.19	0.02	0.02	0.04	0.09	0.22	0.45
Male	19 + Years	7,302	0.18	0.02	0.01	0.03	0.08	0.21	0.44
Male	19 - 50 Years	3,944	0.16	0.02	0.01	003	0.08	0.19	0.39
Male	51 + Years	3,358	0.21	0.03	0.02	0.04	0.10	0.24	0.51
Female	19 + Years	7,036	0.13	0.01	0.01	0.02	0.05	0.14	0.31
Female	19 - 50 Years	3,641	0.10	0.01	0.01	0.01	0.04	0.10	0.23
Female	51 + Years	3,395	0.17	0.02	0.01	0.03	0.08	0.19	0.41

### ALA from foods and dietary supplements

Mean usual intake of ALA from foods alone and foods in combination with dietary supplements was determined for several age groups in adults ≥19 years of age (see Tables 3 and 4). In all adults ≥19 years of age, mean usual intake was 1.5 ± 0.01 g/day with only 59 ± 1.4% above the AI. Mean usual intake of ALA was comparable when considering food and dietary supplement sources in adults ≥ 19 years of age, with 61 ± 2.2% above the AI. In general, males had a greater percentage not meeting the AI in comparison to females.

**Table 3:** Mean usual intake of ALA from foods (g/day) in adults from NHANES 2003–2008

			**Usual intake**		**Percentile**					**Al**	
**Gender**	**Age**	**N**	**Mean**	**SE**	**10**	**25**	**50**	**75**	**90**	**% Above**	**SE**
All	19 + Years	14,338	1.5	0.01	0.9	1.1	1.4	1.8	2.3	59.2	1.4
All	19 - 50 Years	7,586	1.6	0.02	0.9	1.1	1.5	1.9	2.3	61.2	1.7
All	51 + Years	6,753	1.5	0.02	0.8	1.1	1.4	1.8	2.2	56.4	1.2
Male	19 + Years	7,302	1.7	0.02	1.1	1.3	1.7	2.1	2.5	54.4	1.6
Male	19 - 50Years	3,944	1.8	0.03	1.1	1.4	1.7	2.1	2.5	57.8	2.0
Male	51 + Years	3,358	1.6	0.03	1.0	1.2	1.6	2.0	2.4	48.2	1.8
Female	19 + Years	7,036	1.3	0.02	0.8	1.0	1.3	1.6	2.0	64.0	1.7
Female	19 - 50 Years	3,641	1 3	0.03	0.8	1.0	1.3	1.6	2.0	64.7	2.4
Female	51 + Years	3,395	1.3	0.02	0.8	1.0	1.2	1.6	2.0	63.2	1.5

**Table 4:** Mean usual intake of ALA from foods and dietary supplement in adults from NHANES 2003–2008

			**Usual intake**		**Percentile**					**Al**	
**Gender**	**Age**	**N**	**Mean**	**SE**	**10**	**25**	**50**	**75**	**90**	**% Above**	**SE**
All	19 + Years	3,383	1.6	0.04	0.9	1.1	1.5	1.9	2.4	61.2	2.2
All	19-50 Years	2,280	1.6	0.04	0.9	1.1	1.5	1.9	2.4	61.7	2.7
All	51 + Years	1,103	1.6	0.08	0.8	1.1	1.4	1.8	2.3	60.1	2.3
Male	19 + Years	1,566	1.8	0.04	1.0	1.3	1.7	2.1	2.6	54.6	2.8
Male	19-50 Years	1,100	1.8	0.05	1.0	1.3	1.7	2.1	2.6	55.6	3.3
Male	51 + Years	466	1.7	0.06	1.0	1.2	1.6	2.1	2.6	51.2	3.5
Female	19 + Years	1,817	1.4	0.05	0.8	1.0	1.3	1.7	2.1	66.7	3.4
Female	19-50 Years	1,180	1.4	0.05	0.8	1.0	1.3	1.7	2.1	67.1	4.3
Female	51 + Years	637	1.5	0.12	0.8	1.0	1.3	1.7	2.1	65.4	3.1

### DHA and EPA from foods and dietary supplements

Mean usual intake of EPA and DHA from foods alone and foods in combination with dietary supplements were calculated for all adults ≥19 years of age (see Tables 5 and 6 for EPA and Tables 7 and 8 for DHA). Intake from foods alone for DHA and EPA were 63 ± 2 and 23 ± 1 mg/day, respectively. DHA and EPA from food only median intakes were 50 mg/d and 18 mg/d, respectively. Intake of DHA and EPA from foods and dietary supplements was 72 ± 4 mg/d, and 41 ± 4 mg/d, respectively.

**Table 5:** Mean usual intake of EPA from foods (g/day) in adults from NHANES 2003–2008

			**Usual intake**		**Percentile**				
**Gender**	**Age**	**N**	**Mean**	**SE**	**10**	**25**	**50**	**75**	**90**
All	19 + Years	14,338	23	1	7	11	18	29	43
All	19 - 50 Years	7,585	23	1	7	11	18	29	44
All	51 + Years	6,753	22	1	8	12	18	28	42
Male	19 + Years	7,302	27	1	9	14	22	34	51
Male	19 - 50 Years	3,944	28	2	9	14	23	35	52
Male	51 + Years	3,358	26	2	9	13	21	33	49
Female	19 + Years	7,036	18	1	7	10	15	23	34
Female	19 - 50 Years	3,641	18	1	6	9	115	23	33
Female	51 + Years	3,395	19	1	7	11	16	25	36

**Table 6:** Mean usual intake of EPA from foods and dietary supplement in adults from NHANES 2003–2008

			**Usual intake**		**Percentile**				
**Gender**	**Age**	**N**	**Mean**	**SE**	**10**	**25**	**50**	**75**	**90**
All	19 + Years	3,383	41	4	7	11	18	28	45
All	19 - 50 Years	2,280	34	3	7	11	18	28	43
All	51 + Years	1,103	58	9	7	11	18	29	58
Male	19 + Years	1,566	44	6	11	15	22	33	49
Male	19 - 50 Years	1,100	37	5	11	15	23	33	47
Male	51 + Years	466	63	14	10	14	21	33	69
Female	19 + Years	1,817	39	4	6	9	15	24	41
Female	19 - 50 Years	1,180	31	3	6	9	14	23	37
Female	51 + Years	637	55	8	7	10	16	27	54

**Table 7:** Mean usual intake of DHA from foods (g/day) in adults from NHANES 2003–2008

			**Usual intake**		**Percentile**				
**Gender**	**Age**	**N**	**Mean**	**SE**	**10**	**25**	**50**	**75**	**90**
All	19 + Years	14,338	63	2	21	32	50	79	119
All	19 - 50 Years	7,585	63	2	20	31	50	80	120
All	51 + Years	6,753	62	2	21	32	50	78	116
Male	19 + Years	7,302	75	3	25	39	61	95	1140
Male	19 - 50 Years	3,944	77	3	26	40	63	98	145
Male	51 + Years	3,358	71	4	24	37	58	90	132
Female	19 + Years	7,036	51	2	18	27	42	64	94
Female	19 - 50 Years	3,641	48	2	17	26	40	61	89
Female	51 + Years	3,395	54	3	19	29	45	68	100

**Table 8:** Mean usual intake of DHA from foods and dietary supplement in adults from NHANES 2003–2008

			**Usual intake**		**Percentile**				
**Gender**	**Age**	**N**	**Mean**	**SE**	**10**	**25**	**50**	**75**	**90**
All	19 + Years	3,383	72	4	12	20	39	77	157
All	19 - 50 Years	2,280	68	4	11	20	38	75	147
All	51 + Years	1,103	81	6	12	21	40	82	188
Male	19 + Years	1,566	90	7	13	25	50	103	197
Male	19 - 50 Years	1,100	87	8	14	25	50	99	185
Male	51 + Years	466	94	12	12	23	48	105	234
Female	19 + Years	1,817	59	4	11	18	33	61	121
Female	19 - 50 Years	1,180	53	4	10	17	31	58	108
Female	51 + Years	637	72	8	12	20	36	69	154

Males also had higher (p < 0.05) intake of EPA and DHA from foods and dietary supplements as compared to females (44 ± 6 vs. 39 ± 4 and 90 ± 7 vs. 59 ± 4 mg/d, respectively) and older adults had higher intakes of EPA, but not DHA as younger adults (EPA: 34 ± 3 vs. 58 ± 9, p < 0.05; DHA: 68 ± 4 vs. 81 ± 6, p < 0.05).

## Discussion

In general, the present NHANES analysis demonstrates that a large percentage of the US adult population is not meeting recommendations for omega-3 fatty acid consumption set forth by the 2010 DGA. Intakes of fish high in omega-3 fatty acids EPA and DHA, were greater in older adults and in males in comparison to younger adults and females, respectively.

Heart disease is the leading cause of death for both men and women in the US [1]. The 2010 Report of the Dietary Guidelines Advisory Committee (DGAC) on the Dietary Guidelines for Americans acknowledged that Americans adults consume too little seafood and should be encouraged to increase consumption to leverage heart health benefits [19]. The DGAC cited previously published literature that demonstrated biological effects of EPA and DHA. Specifically, EPA and DHA supplementation as a treatment strategy lowered blood concentration of triacylglycerol as a marker of CVD, lowered overall mortality in persons with CVD, and lowered arrhythmias and sudden death [19,20]. This prompted the 2010 DGA to recommend 8 oz of seafood per week to contribute an average of 250 mg per day of long-chain omega-3 fatty acids, for all Americans. Furthermore, 2010 DGA [5] cited the importance of ensuring maternal dietary intake of long chain omega-3 fatty acids, in particular DHA, during pregnancy and lactation. The American Heart Association’s recommendation is to consume at least two 3.5 oz fish meals per week to reduce the risk of CVD, with an emphasis on fatty fish (i.e., salmon, herring, mackerel, sardines) to increase EPA and DHA [6]. A total of 1 gram per day of EPA plus DHA from a combination of higher omega-3 fatty acid- containing fish and supplements, if needed, in individuals with established CVD [3,5,6].

Fish is not a habitually consumed food in the US, creating a challenge in estimating usual intake [7]. In the US, per capita salmon consumption represents the single largest contributor to dietary intake of long-chain omega-3 fatty acids [21]. Previous findings report intake of total omega-3 fatty acids in the United States to be approximately 1.6 g/day, of which 0.1-0.2 g/day stemming from EPA and DHA and 1.4 g/day from ALA [22]. Our current data show that US adults ≥ 19 years of age consume 0.41 g/day and 0.72 g/day of EPA and DHA from foods and supplements, respectively. While daily intake has increased substantially in nearly two decades, American adults are not meeting recommendations for fish-derived omega-3 fatty acids. Interestingly, our study showed comparable ALA intake to the earlier study [22], suggesting that plant-based omega-3 fatty acids may not have the consumer awareness when it pertains to heart health benefits.

Both recent and previously published literature, including evidence from randomized controlled trials, have documented the cardiovascular benefits linked to dietary omega-3 fatty acid consumption in CVD patients as well as healthy individuals [3,7,23-25]. While CVD is a leading cause of death in Americans, the disease rarely manifests in childhood or adolescence [26], however, the process begins in childhood and can be highly reversible (see [27] for review). In contrast, compelling evidence supports that early identification of predisposing factors and lifestyle modifications can significantly reduce the incidence of clinical disease development [26]. Children do not develop atherosclerosis per se, but rather present fatty streaks that are reversible (see [27] for review). While long-chain omega-3 fatty acid consumption benefits are not well established in children, as they are in adults, preliminary evidence suggests cardiovascular benefits in children, including improved endothelial function [28] and blood pressure [29]. In fact, when considering blood pressure, researchers have suggested that elevated blood pressure in adulthood may be associated with perinatal omega-3 fatty acid deficiency [29]. Again, such studies suggest that early exposure to dietary long-chain omega-3 are play a critical role in supporting heart health and reducing CVD risk in later life.

A limitation of this report is that the estimates relied on self-reported dietary data for intake of total fish and omega-3 fatty acids from both foods and dietary supplements. The models that we applied also relied on assumptions that reported nutrient intakes from food sources on 24-h recalls were unbiased, and the self-reported dietary supplement intake reflected the true long-term supplement intake. The data presented in the manuscript should also be interpreted ones that provide associations and not cause and effect due to the observational nature of the analysis.

## Conclusion

Our current observational findings show that a significant number of American adults are not meeting recommendations for omega-3 fatty acid intake. This dietary behavior may have negative consequences to CVD risk. CVD builds over a lifetime, with initiation and progression commencing during the pediatric years, strengthening the argument to focus on nutrition behavior and select food consumption during childhood. As we are approaching the development and release of the 2015 DGA, specific strategies to increase consumption of omega-3 fatty acids in the U.S. population need to be addressed. As omega-3 fatty acids are deemed important from authoritative bodies, a collaborative strategy of dietary supplementation (i.e., fish oil supplements), food fortification, in addition to food sources (i.e., fish consumption) may need to be considered to achieve recommendations in the American population and to have significant and beneficial public health impact.

## Competing interests

Financial interest statement: This project has been funded by Pharmavite, LLC.

Conflict of interest statement: YP as Vice President of Nutritional Strategies Inc provides food, nutrition and regulatory affairs consulting services for numerous food and beverage companies and food-related associations and collaborates with VLF on NHANES analyses; VLF as Senior Vice President of Nutrition Impact, LLC provides food and nutrition consulting services for numerous food and beverage companies. VLF also conducts analyses of NHANES data for members of the food industry. Both JB and CR are currently employed with Pharmavite, LLC, which provided funding for the current analyses.

## Authors’ contributions

YP collaborated on the interpretation and drafted the manuscript; VLF designed the research, conducted analyses and provided interpretation; JB and CR contributed to the manuscript; all authors read and approved the final manuscript.
