# Correction: U.S. adults are not meeting recommended levels for fish and omega-3 fatty acid intake: results of an analysis using observational data from NHANES 2003–2008

**DOI:** 10.1186/1475-2891-13-64

**Published:** 2014-06-25

**Authors:** Yanni Papanikolaou, James Brooks, Carroll Reider, Victor L Fulgoni

**Affiliations:** 1 Nutritional Strategies Inc, 59 Marriot Place, Paris, ON N3LOA3, Canada; 2 Pharmavite, LLC, Northridge, CA, USA; 3 Pharmavite, LLC, Mission Hills, CA, USA; 4 Nutrition Impact, LLC, Battle Creek, MI, USA

## Correction

Following the publication of this article [1], we noted errors to Tables five and seven (Tables 1 and 2 here). Corrected versions are presented below.

**Table 1:** Mean usual intake of EPA from foods (mg/day) in adults from NHANES 2003–2008

			**Usual Intake**		**Percentile**				
**Gender**	**Age**	**N**	**Mean**	**SE**	**10**	**25**	**50**	**75**	**90**
All	19 + Years	14,338	23	1	7	11	18	29	43
All	19–50 + Years	7,585	23	1	7	11	18	29	44
All	51 + Years	6,753	22	1	8	12	18	28	42
Male	19 + Years	7,302	27	1	9	14	22	34	51
Male	19–50 + Years	3,944	28	2	9	14	23	35	52
Male	51 + Years	3,358	26	2	9	13	21	33	49
Female	19 + Years	7,036	18	1	7	10	15	23	34
Female	19–50 + Years	3,641	18	1	6	9	15	23	33
Female	51 + Years	3,395	19	1	7	11	16	25	36

**Table 2:** Mean usual intake of DHA from foods (mg/day) in adults from NHANES 2003–2008

			**Usual Intake**		**Percentile**				
**Gender**	**Age**	**N**	**Mean**	**SE**	**10**	**25**	**50**	**75**	**90**
All	19 + Years	14,338	63	2	21	32	50	79	119
All	19–50 + Years	7,585	63	2	20	31	50	80	120
All	51 + Years	6,753	62	2	21	32	50	78	116
Male	19 + Years	7,302	75	3	25	39	61	95	140
Male	19–50 + Years	3,944	77	3	26	40	63	98	145
Male	51 + Years	3,358	71	4	24	37	58	90	132
Female	19 + Years	7,036	51	2	18	27	42	64	94
Female	19–50 + Years	3,641	48	2	17	26	40	61	89
Female	51 + Years	3,395	54	3	19	29	45	68	100

We also noted that the third paragraph of the Discussion states that our data shows daily consumption of EPA and DHA of 0.41 g and 0.72 g, respectively, however this should be 41 mg/day and 72 mg/day, respectively.

Lastly, the titles of Tables six and eight in the published manuscript should be displayed as, “Mean usual intake of EPA from foods and dietary supplements (mg/day) in adults from NHANES 2003-2008” and “Mean usual intake of DHA from foods and dietary supplements (mg/day) in adults from NHANES 2003-2008”, respectively.
